# Ameliorative effects of Gualou Guizhi decoction on inflammation in focal cerebral ischemic-reperfusion injury

**DOI:** 10.3892/mmr.2015.3515

**Published:** 2015-03-19

**Authors:** YUQIN ZHANG, SHENGNAN ZHANG, HUANG LI, MEI HUANG, WEI XU, KEDAN CHU, LIDIAN CHEN, XIANWEN CHEN

**Affiliations:** 1 College of Pharmacy Fujian University of Traditional Chinese Medicine, Fuzhou, Fujian 350122, P.R. China; 2 College of Rehabilitation Medicine, Fujian University of Traditional Chinese Medicine, Fuzhou, Fujian 350122, P.R. China

**Keywords:** Gualou Guizhi decoction, focal cerebral ischemic-reperfusion injury, cytokines, nuclear factor-κB

## Abstract

Gualou Guizhi decoction (GLGZD) is a well-established Traditional Chinese Medicinal formulation which has long been used to treat stroke in a clinical setting in China. The present study investigated the ameliorative effects of GLGZD on inflammation in focal cerebral ischemic-reperfusion injury. A rat model of middle cerebral artery occlusion (MCAO) was employed. Rats were administrated GLGZD (7.2 and 14.4 g/kg per day) or saline as control 2 h after reperfusion and daily over the following seven days. Neurological deficit score and screen test were evaluated at 1, 3, 5 and 7 days after MCAO. Brain infarct size and brain histological changes were observed via 2,3,5-triphenyltetrazolium chloride staining and regular hematoxylin & eosin staining. Furthermore, inflammation mediators and nuclear factor-κB (NF-κB) were investigated using ELISA and immunohistochemistry. GLGZD treatment significantly improved neurological function, ameliorated histological changes to the brain and decreased infarct size in focal cerebral ischemic-reperfusion injury. GLGZD was found to significantly reduce interleukin (IL)-1, tumor necrosis factor-α and NF-κB levels, while increasing levels of IL-10. In conclusion, the present study suggested that GLGZD has a neuroprotective effect on focal cerebral ischemic-reperfusion injury and this effect is likely to be associated with the anti-inflammatory function of GLGZD.

## Introduction

Stroke is a common refractory disease, which is a serious hazard to human health and safety. Stroke is associated with high incidence, high morbidity and high mortality (1). Ischemic stroke accounts for ~80% of cases of stroke. Stroke is the third leading cause of mortality in the USA and Great Britain, with an incidence of up to 0.2% of the population every year (2,3) following heart disease and cancer. According to statistics, there are ~200 million stroke patients each year in China, of which 70–80% lose their independence due to disability (4). Stroke causes a significant public health concern due to the suffering caused as a result of a stroke and also the economic burden. However, to date, therapies for ischemic stroke remain limited; therefore, there is a requirement for novel, effective and widely applicable pharmacological treatments.

The pathophysiology of stroke is associated with multiple complex factors, including excitotoxicity, oxidative stress, inflammation or apoptosis (5). There are different mechanisms involved in the pathogenesis of stroke and in particular, inflammation has a central role (6–8). Inflammatory responses involving the production of inflammatory cytokines and infiltration into the brain of inflammatory cells are initiated subsequent to ischemic injury. This has been observed during ischemic pathology in experimental animal models of stroke as well as in humans (9,10). Inflammation is hypothesized to exacerbate brain damage due to microglial activation, resident perivascular and parenchymal macrophages as well as infiltration of peripheral inflammatory cells. Cytokines, including interleukin (IL)-1β, IL-6, tumor necrosis factor (TNF)-α, IL-10 and transforming growth factor-β (TGF-β) are associated with inflammation in ischemic stroke (11–13). TNF-α is an important regulatory factor in the body’s inflammatory and immune response; TNF-α is able to promote the inflammatory response and its inhibition reduces ischemic brain injury (14).

IL-1 is a cytokine involved in host defense. It is predominantly involved in the immune response, inflammation, fever and acute phase protein synthesis. Systemic or locally-induced IL-1 can effectively start, strengthen or prolong the inflammatory response. Changes in vascular endothelial cells corresponding to IL-1 function allow for leukocyte infiltration into the area of inflammation. IL-1 is increased following permanent or transient cerebral ischemia and can stimulate the activation and proliferation of astrocytes and glial cells (15). It mediates major histocompatibility complex antigens on the astrocytic surface and stimulates the secretion of IL-6 by astrocytes, which leads to astrocytic amplification, myelin damage and oligodendrocyte injury. IL-6 is pleiotropic and is expressed at a high rate following stroke (16); however, it remains to be elucidated whether IL-6 is pro-inflammatory, anti-inflammatory, or both. IL-10 is an anti-inflammatory cytokine and suppresses IL-1 and TNF-α production. NF-κB is a pleiotropic transcription factor and inactive in quiescent cells. When it is activated, it can promote the transcription of a variety of certain genes, releasing a series of cytokines to contribute to inflammation. An excessive inflammatory reaction can also damage target cells and tissue.

At present, there are numerous treatments for ischemic stroke in the clinic (17), including etiological treatment, conventional medical treatment, drug treatment, neuro-interventional therapy, stem cell transplantation as well as acupuncture and massage therapy. However, there remains a lack of effective therapies. At present, drug treatment is the main method to treat ischemic stroke. Western medicine for stroke is generally divided into the following categories: Thrombolytic drugs (18), anti-platelet drugs (19), defibrase drugs (20), anti-coagulant drugs and neuroprotective drugs (21–23). Ischemic stroke is a multi-factor, multi-mechanism, multi-link cascade; therefore, it cannot be effectively treated with only one drug or therapy. Traditional Chinese Medicinal formulations have certain characteristics which make them suitable targets for investigation, including their safety with less adverse side-effects, abundant resources of certain plants, low cost and extensive effects. There is important practical significance in examining anti-ischemic stroke drugs from the rich resource of Traditional Chinese Medicine. GLGZD, a well-established Traditional Chinese Medicinal formula, is composed of six herbs, including *Trichosanthis* radix, *Ramulus cinnamomi, Paeonia lactiflora, Glycyrrhiza, Zingiber officinale* Roscoe and *Fructus jujubae*, according to the yin-yang and Wu Hsing (five elements) theories of Traditional Chinese Medicine. It has been used clinically to treat muscular spasticity following stroke, epilepsy or spinal cord injury in China (24–26). Preliminary studies by our group showed beneficial effects of GLGZD in stroke patients (unpublished data). Previously, the decoction’s effects were evaluated on lipopolysaccharide-induced BV-2 murine microglial cells, which indicated that GLGZD had an effect on the toll-like receptor-4/NF-κB pathway (27). However, its underlying mechanism with regard to its anti-inflammatory effects remains to be elucidated. In the present study, a rat model of cerebral ischemia - middle cerebral artery occlusion (MCAO) was established to investigate the potential effects of GLGZD on focal cerebral ischemic-reperfusion injury. The possible mechanism associated with downregulation of TNF-α, ILs and NF-κB in GLGZD-treated rats was also examined.

## Materials and methods

### Animals and materials

A total of 44 specific-pathogen free male Sprague-Dawley (SD) rats, weighing 180–220 g and aged two months, were provided by the Laboratory Animal Center of Fujian University of Traditional Chinese Medicine (Fuzhou, China). The animals were housed under controlled temperature (21–23°C), relative humidity 55±5%, a 12-h light/dark cycle and had free access to a standard rat diet and tap water. Animal treatments were strictly in accordance with International Ethics Guidelines and the National Institutes of Health Guidelines Concerning the Care and Use of Laboratory Animals, and the experiments were approved by the Institutional Animal Care and Use Committee of Fujian University of Traditional Chinese Medicine (Fuzhou, China).

The medicinal herbs were purchased from Tongchun drugstore (Fuzhou, China) and they were identified by Professor Yang (Pharmaceutical college, Fujian University of Traditional Chinese Medicine, Fujian, China). Standard substances (peoni-florin, liquiritigenin, liquiritin, cinnamic acid, cinnamaldehyde and glycyrrhizic acid) were purchased from the National Institute for the Control of Pharmaceutical and Biological Products (Beijing, China). Acetonitrile was high performance liquid chromatography (HPLC) grade and purchased from Merck KGaA (Darmstadt, Germany). Deionized water used throughout the experiments was generated using a Millipore water purification system (Milli-Q^®^ Direct-Q 3; Millipore, Milford, MA, USA). All other chemicals used, unless otherwise stated, were obtained from Sinopharm Chemical Reagent Co., Ltd (Shanghai, China) or Sigma-Aldrich (Shanghai) Co. LLC. (Shanghai, China).

### Preparation and HPLC analysis of GLGZD

GLGZD was prepared from the six herbs with the ratio of 10:3:3:3:2:3 (dry weight; in the aforementioned order) and extracted with 80% ethanol twice for 1 h each time. The filtrate was recovered from the ethanol and concentrated to achieve a solution with a relative density of 1.2 (50°C). The decoction was obtained for further use.

The above preparation was passed through a 0.45-*μ*m nylon filter prior to being subjected to HPLC fingerprinting analysis, where major peaks were identified as the characteristic active components of the individual herbs by comparison with chromatograms of reference compounds (28). The HPLC system (Shimadzu, Kyoto, Japan) was equipped with an LC-20A pump system, photodiode array detector SPD-M20A and a diamonsil C18 reverse-phase column (I.D. 4.6×250 mm, 5 *μ*m). Separation was achieved with a linear gradient program using mobile phase A (acetonitrile) and mobile phase B (water containing 0.1% phosphoric acid). Elution was started with a gradient of 95% B changing to 68% over 45 min and finally to 52% B over 15 min, then remaining at 52% B for 5 min. Flow rate and injection volume were 1.0 ml/min and 10 *μ*l, respectively. Analysis was performed in triplicate.

### Focal cerebral ischemia-reperfusion model and drug treatments

The focal cerebral ischemia-reperfusion model was generated as described previously (28). Briefly, rats were anesthetized, then the common left carotid artery (CCA), the external carotid artery (ECA) and the internal carotid artery (ICA) were exposed by a 3–0 surgical monofilament nylon suture with a rounded tip (Guangzhou Jialing Biotechnology Co., Ltd., Guangzhou, China), which was carefully inserted from the ECA into the ICA and was advanced towards to occlude the origin of the left middle cerebral artery (MCA) until a light resistance was felt (~18–22 mm). After 2 h of occlusion, the nylon suture was withdrawn for blood reperfusion. Sham-operated rats underwent the same procedure but without focal cerebral ischemia-reperfusion. During surgical procedures, rat body temperature was maintained at 37±0.5°C. Following surgery, the rats were allowed to recover in pre-warmed cages.

Rats with successfully established focal cerebral ischemia-reperfusion were divided into four experimental groups and all rats were grouped as either the sham-operated group, the model group, the GLGZD-1 group or the GLGZD-2 group. GLGZD was administered at doses of 7.2 g/kg (GLGZD-1 group) and 14.4 g/kg (GLGZD-2 group) 2 h after reperfusion followed by daily administration for seven subsequent days. A 0.9% saline solution was used in the sham-operated group.

### Neurological score

The neurological score of rats was assessed according to the criteria of a five-point scale (20), whereby a score of 0 indicates no neurological symptoms, 1 indicates inability to fully flex the left forepaw, 2 indicates rotating while crawling and falling to the contralateral side, 3 indicates inability to walk unaided and 4 indicates unconsciousness. Behavioral tests were performed in rats after 60 min of reperfusion followed by 7 days of treatment with GLGZD. The evaluation was performed by an observer who was blinded to the experimental group.

### Cerebral infarct volume measurement

A total of six rats in each group were sacrificed by decapitation and the intact brain was removed on the 7th day after MCAO. The brain was cut into six coronal sections following being maintained at −20°C for ~10 min and stained with 2% 2,3,5-triphenyltetrazolium chloride (TTC) solution in phosphate buffer (pH 7.4). The sections were maintained at 37°C for 1 h and turned over several times. Following staining, the infarct size was calculated as a percentage fraction of non-viable cerebral tissue of the global brain.

### ELISA

A total of eight rats in each group were sacrificed by anesthetization with 10% chloral hydrate solution (0.3 ml/100 g, i.p.) and blood was obtained from the abdominal aorta. Subsequently, blood samples were centrifuged at 628 x g for 20 min to obtain serum. Levels of IL-1β, IL-6, IL-10 and TNF-α were measured using ELISA kits (IL-1β ELISA kit L120530583; USCN Life Science Inc., Houston, TX, USA; IL-6 ELISA kit 133897105, IL-10 ELISA kit 1388127105; Wuhan Boster Biological Technology, Ltd., Wuhan, China; TNF-α; Ameko, Shanghai, China) in serum according to the manufacturer’s instructions.

### Histological examination

A total of six rats in each group were anesthetized and fixed with a PBS-buffered 4% parafor-maldehyde solution (Sinopharm Chemical Reagent Co., Ltd) by transcardial perfusion. Subsequently, the intact brain was removed and embedded with paraffin (Beijing Zhongshan Golden Bridge Biotechnology Co., Ltd, Beijing, China). Paraffin-embedded sections were cut into 5-*μ*m slices. Standard hematoxylin & eosin staining was performed and histo-pathological changes were observed under a light microscope (DM4000B; Leica Microsystems GmbH, Wetzlar, Germany).

### Immunohistochemistry

Paraffin-embedded sections were used for assessment of TNF-α and NF-κB expression. Briefly, paraffin sections were dewaxed and boiled, at ~95°C for 15 min, in citrate buffer (Beijing Zhongshan Golden Bridge Biotechnology Co., Ltd) for antigen retrieval. Subsequently, sections were incubated with the primary antibodies at room temperature for 2 h, followed by incubation in 3% H_2_O_2_ (Sinopharm Chemical Reagent Co., Ltd) and blocking with normal goat serum. Sections were then rinsed in PBS and incubated with rabbit polyclonal anti-TNF-α (1:500; bs-0078R) or rabbit polyclonal anti-NF-κB (p65) antibody (1:500; bs-0465R) (Beijing Biosynthesis Biotechnology Co., Ltd, Beijing, China), respectively, overnight at 4°C. Sections were then incubated with a biotinylated secondary antibody (polyclonal goat anti-rabbit IgG/streptavidin antibody, Beijing Zhongshan Golden Bridge Biotechnology Co., Ltd; 1:500, 37°C for 2 h), washed and incubated with horseradish peroxidase-labeled streptavidin. Finally, slides were developed with diaminobenzidine and counterstained with hematoxylin. The primary antibody was omitted in the negative control. Microscopic images were acquired using a Leica microscope (DM4000B; Leica Microsystems GmbH) and five high-power fields (magnification, ×400) were randomly selected in each slide and the average proportion of positive cells in each field were counted by the true color multi-functional cell image analysis management system (Image-Pro Plus; Media Cybernetics, Minneapolis, MN, USA).

### Statistical analysis

All values are expressed as the mean ± standard deviation. The statistical significance of differences between two groups was determined by Student’s t-test. For multiple group comparisons, values were analyzed by one-way analysis of variance. Statistical analyses were performed using SPSS 16.0 (SPSS, Inc., Chicago, IL, USA). P<0.05 was considered to indicate a statistically significant difference.

## Results

### Quality control of GLGZD

**Figure 1:**
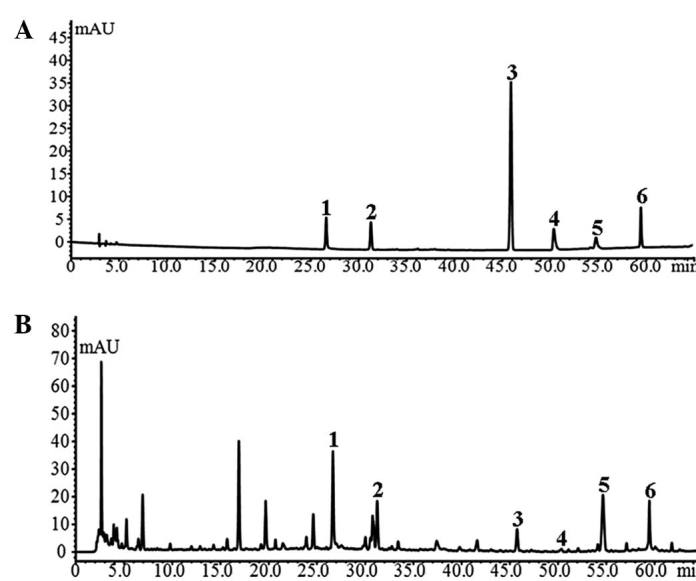
Liquid chromatograms of standard and sample. (A) Liquid chro-matogram of the standard, where 1 is peoniflorin, 2 is liquiritigenin, 3 is liquiritin, 4 is cinnamic acid, 5 is cinnamaldehyde and 6 is glycyrrhizic acid. (B) Liquid chromatogram of Gualou Guizhi decoction.

GLGZD is a well-known Traditional Chinese Medicinal formulation. An optimal procedure for preparing the GLGZD extract was established as mentioned above. Reference standard samples of peoniflorin, liquiritigenin, liquiritin, cinnamic acid, cinnamaldehyde and glycyrrhizic acid were selected as quality control indexes. Fingerprint chromatograms of GLGZD and standard samples are shown in Fig. 1. The chromatogram of GLGZD showed a satisfactory peak separation and was adjusted to the separation requirement.

### GLGZD reduces brain damage following ischemic brain injury

**Figure 2:**
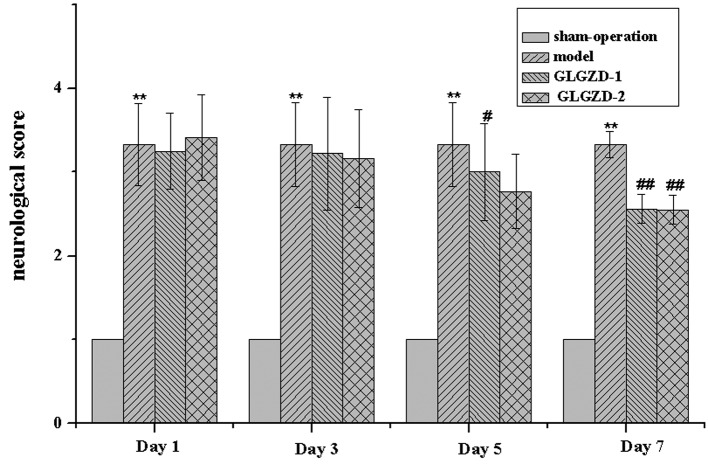
Effect of GLGZD on the development of behavioral abnormalities following MCAO. Neurological score was taken as index. Values are expressed as the mean ± standard deviation in each group. 0 indicates no neurological symptoms, 1 indicates inability fully flex the left forepaw, 2 indicates rotating while crawling and falling to the contralateral side, 3 indicates inability to walk unaided and 4 indicates unconsciousness. ^**^P<0.01 vs. sham-surgery group, ^#^P<0.05, ^##^P<0.01, vs. MCAO model group. MCAO, middle cerebral artery occlusion; GLGZD, Gualou Guizhi decoction; GLGZD-1, rats that were administered 7.2 g/kg GLGZD; GLGZD-2, rats that were administered 14.4 g/kg GLGZD.

Neurological deficits, cerebral infarct size and brain histology were evaluated to investigate whether GLGZD could reduce brain injury in rats following MCAO. A neurological behavioral scoring system was used to evaluate neurological deficits. Neurological deficit scores observed in the model group were 3.33±0.49 at day 1, 3.33±0.50 at day 3, 3.33±0.49 at day 5 and 3.33±0.16 at day seven, respectively. Of note, the neurological deficit scores were lower in the GLGZD groups, indicating improved neurological function, with lower scores at day 5 (3.00±0.58 and 2.77±0.44; P<0.05) and day 7 (2.56±0.17 and 2.55±0.17; P<0.01; Fig. 2).

**Figure 3:**
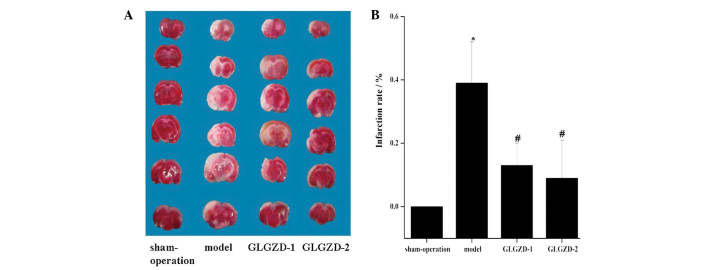
Effect of GLGZD on cerebral infarction in MCAO model rats. (A) Representative 2,3,5-triphenyltetrazolium chloride-stained brain coronal sections of the four groups. The normal tissue was stained to red-blue and the infarct area was stained to pale pink. (B) Infarction rate was quantified using the Motic Med 6.0 system. Values are expressed as the mean ± standard deviation from five individual rats in each group. ^*^P<0.05, vs. sham-surgery group, ^#^P<0.05, vs. model group. MCAO, middle cerebral artery occlusion; GLGZD, Gualou Guizhi decoction; GLGZD-1, rats that were administered 7.2 g/kg GLGZD; GLGZD-2, rats that were administered 14.4 g/kg GLGZD.

Brain cerebral infarct size was evaluated using TTC staining and the viable cerebral tissue was stained red while the infarcted cerebral tissue remained pale. GLGZD signifi-cantly decreased the infarct size as compared with that of the model group at day 7 following ischemia (Fig. 3). The infarction rates in the GLGZD-1, GLGZD-2, model and sham groups were 0.16±0.03 (P<0.05 vs. model), 0.13±0.01 (P<0.05 vs. model), 0.39±0.02 (P<0.05 vs. sham) and 0.09±0.02 at 7 days, respectively (Fig. 3).

**Figure 4:**
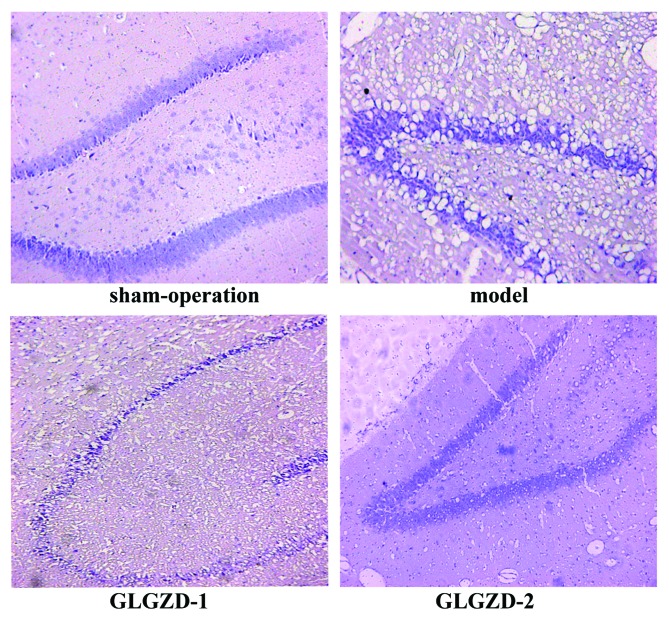
Effect of GLGZD on the histopathological changes in brain tissue following MCAO or treated with GLGZD. Representative images are shown (hematox-ylin and eosin stained; magnification, ×200). MCAO, middle cerebral artery occlusion; GLGZD, Gualou Guizhi decoction; GLGZD-1, rats that were administered 7.2 g/kg GLGZD; GLGZD-2, rats that were administered 14.4 g/kg GLGZD.

Brain histological analysis was performed using hematoxylin & eosin staining. As shown in Fig. 4, in the sham-operated group, the brain tissues remained intact, neurons remained eumorphic and maintained a uniform distribution, the cytoplasm was pale pink and abundant and no inflammatory cells were observed to have infiltrated. By contrast, in the MCAO group, the brain tissues had a larger infarct area. Neuronal arrangement became disorderly and decreased. Rats in the MCAO group exhibited an increased volume and the cytoplasm exhibited pale staining. In addition, in certain areas of the cytoplasm, hypervacuolization was observed. However, following treatment with GLGZD, the infarct area was markedly reduced, the extent of damage was significantly diminished and cytoplasmic hypervacuolization was decreased.

### GLGZD on attenuates inflammatory response in ischemic brain injury

Inflammatory mediators, including IL-1β, IL-6, IL-10, TNF-α and NF-κB were assessed to examine whether GLGZD was involved in the inflammatory response in ischemic brain injury. Table I shows that cerebral ischemia/reperfusion significantly increased the levels of IL-1β and IL-10 in the serum. GLGZD treatment decreased the levels of IL-10 and IL-1β as compared with those in the model group (P<0.05). By contrast, the levels of IL-6 did not demonstrate a difference between the model and the sham-surgery group and increased following GLGZD treatment.

**Figure 5:**
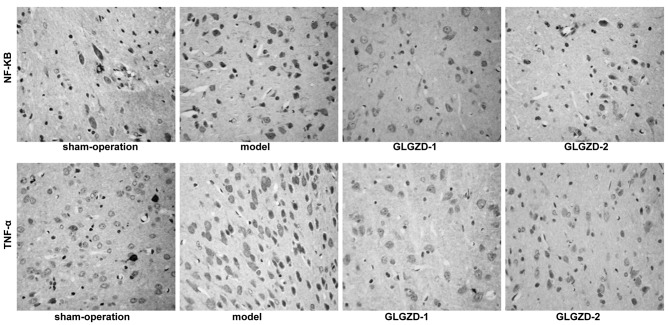
Immunohistochemistry for NF-κB and TNF-α in the brain of rats following MCAO or treated with GLGZD. Representative images are shown (magnification, ×100). The positive cells were stained dark. MCAO, middle cerebral artery occlusion; GLGZD, Gualou Guizhi decoction; NF, nuclear factor; TNF, tumor necrosis factor; GLGZD-1, rats that were administered 7.2 g/kg GLGZD; GLGZD-2, rats that were administered 14.4 g/kg GLGZD.

Levels of NF-κB and TNF-α in the brain were detected by immunohistochemistry. In the model group, the expression of the NF-κB p65 subunit and TNF-α as increased compared with that in the sham-surgery group, but was reduced in GLGZD-treated rats (Fig. 5). As shown in Table II, the immunohistochemical score of NF-κB p65 and TNF-α in the GLGZD-1 group decreased from 5.6±0.4 to 4.8±0.5 and from 5.2±0.7 to 4.6±0.2, respectively (P>0.05). Furthermore, the immunohistochemical score of NF-κB p65 and TNF-α in the GLGZD-2 group decreased significantly from 5.6±0.4 to 4.4±0.6 (P<0.05) and from 5.2±0.7 to 4.4±0.7 (P<0.01), respectively.

In conclusion, these findings suggested that GLGZD attenuated the inflammatory response by regulating inflammatory mediators (IL-1β, IL-6, IL-10 and TNF-α) and NF-κB (p65) against ischemic injury.

## Discussion

In the present study, the effects of GLGZD and its possible inflammatory mechanisms were examined. The inflammatory response partly accounts for the pathogenic progression of ischemic brain injury and involves inflammatory cells and inflammatory mediators (29). It is well-established that cytokines are a group of small glycoproteins (~25 kDa), which are regarded as mediators for regulating the innate and adaptive immune systems. They are produced in response to an activating stimulus and affect cell behavior via specific receptor binding. The main cytokines associated with inflammation in ischemic brain injury are IL-1, TNF-α, IL-6, IL-10 and TGF-β and have been observed to be upregulated in ischemic brain injury (30). The present study confirmed that IL-1β and TNF-α were induced by ischemia; however, IL-1β was downregulated in the serum of the GLGZD-2 group following treatment (P<0.05) and TNF-α was downregulated in the serum of the GLGZD-1 group following treatment (P<0.05). This was consistent with the findings of previous studies (31,32). IL-6 is usually considered a pro-inflammatory cytokine, but in the present study, IL-6 was upregulated following administration of GLGZD particularly at a high dose. Certain studies have shown that IL-6 is an anti-inflammatory cytokine, which may explain the findings of the present study (33,34). At present, it remains to be elucidated as to whether IL-6 is pro-inflammatory or anti-inflammatory, or even both. As an anti-inflammatory cytokine, IL-10 can act by inhibiting IL-1 and TNF-α, and suppressing cytokine receptor expression as well as receptor activation. The present study revealed that the expression of IL-10 in the serum was significantly increased in response to ischemic brain injury, while it was markedly decreased by GLGZD treatment. The most common form of NF-κB, a dimeric transcription factor, is a heterodimer composed of Rel A (p65), namely NF-κB (p65). It is involved in the regulation of inflammation and its activation is correlated with significant increases in levels of IL-1β and TNF-α following cerebral ischemia-reperfusion (35,36). The results of the present study showed that ischemic brain injury markedly induced NF-κB (p65) activation in brain tissue alongside increases in TNF-α levels, while GLGZD markedly suppressed this response. This finding was consistent with results of previous studies (unpublished data).

Administration of GLGZD resulted in potentially neuro-protective effects on ischemic brain injuries induced by MCAO. The behavioral tasks adopted in the present study were designed to assess impairments of the rat brain. Neurological deficit scores were significantly reduced in GLGZD-treated rats. In addition, GLGZD reduced the infarction in the ischemic brain and ameliorated the histological changes in the brain tissue. In conclusion, the present study suggested that GLGZD had a neuroprotective effect in ischemic brain injury, which was due to the inhibition of the inflammatory response and of NF-κB (p65) activation. With the increasing understanding of its mechanism of action and clinical verification, GLGZD may be a potential therapeutic agent for ischemic brain injuries.

## Figures and Tables

**Table I:** Effect of GLGZD on IL-1β, IL-6, IL-10 and TNF-α levels in serum following MCAO.

Group	IL-1β (pg/ml)	IL-6 (pg/ml)	IL-10 (pg/ml)	TNF-α (pg/ml)
Sham-surgery	11.894±1.12	47.258±3.99	11.428±4.90	57.425±16.30
Model	25.822±2.23^a^	41.857±11.25^a^	113.490±21.29^b^	63.187±16.02^a^
GLGZD-1	24.200±2.26	62.810±9.29^d^	55.063±13.30^d^	61.341±6.04^c^
GLGZD-2	19.430±2.11^c^	158.113±30.75^d^	38.895±4.90^d^	63.081±9.03

Values are expressed as the mean ± standard deviation.

a P<0.05,

b P<0.01 vs. sham-surgery group;

c P<0.05,

d P<0.05 vs. MCAO model group. IL, interleukin; TNF, tumor necrosis factor; MCAO, middle cerebral artery occlusion; GLGZD, Gualou Guizhi decoction; GLGZD-1, rats that were administered 7.2 g/kg GLGZD; GLGZD-2, rats that were administered 14.4 g/kg GLGZD.

**Table II:** Comparison of immunohistochemical score of NF-κB and TNF-α in brain tissue.

Group	NF-κB	TNF-α
Sham-surgery	3.2±0.5	1.8±0.4
Model	5.6±0.4^a^	5.2±0.7^b^
GLGZD-1	4.8±0.5	4.6±0.2
GLGZD-2	4.4±0.6^c^	4.4±0.7^d^

a P<0.05,

b P<0.01, vs. sham-surgery group;

c P<0.05,

d P<0.01, vs. middle cerebral artery occlusion model group. TNF, tumor necrosis factor; GLGZD, Gualou Guizhi decoction; NF, nuclear factor; GLGZD-1, rats that were administered 7.2 g/kg GLGZD; GLGZD-2, rats that were administered 14.4 g/kg GLGZD.

## Abbreviations

GLGZD: Gualou Guizhi decoction
MCAO: middle cerebral artery occlusion
TTC: 2,3,5-triphenyltetrazolium chloride
NF-κB: nuclear factor κB
IL: interleukin
TNF: tumor necrosis factor
TGF-β: transforming growth factor-β
